# Acute Pericarditis after Percutaneous Coronary Intervention: A Case Report

**DOI:** 10.3390/medicina57050490

**Published:** 2021-05-13

**Authors:** Greta Rodevič, Povilas Budrys, Giedrius Davidavičius

**Affiliations:** 1 Faculty of Medicine, Vilnius University, M. K. Čiurlionio g. 21/27, LT-03101 Vilnius, Lithuania; greta.rodevic@gmail.com; 2 Clinic of Cardiac and Vascular Diseases, Faculty of Medicine, Vilnius University, Santariškių 2, LT-08661 Vilnius, Lithuania; giedrius.davidavicius@santa.lt; 3 Cardiology and Angiology Center, Vilnius University Hospital Santaros Klinikos, Santariškių 2, LT-08661 Vilnius, Lithuania

**Keywords:** post-cardiac injury syndrome, pericarditis, percutaneous coronary intervention

## Abstract

Background: Percutaneous coronary intervention (PCI) is known as a very rare possible trigger of pericarditis. Most frequently it develops after a latent period or early in the case of periprocedural complications. In this report, we present an atypical early onset of pericarditis after an uncomplicated PCI. Case Summary: A 58-year-old man was admitted to the hospital for PCI of the chronic total occlusion of the left anterior descending (LAD) artery. An initial electrocardiogram (ECG) was unremarkable. The PCI attempt was unsuccessful. There were no procedure-related complications observed at the end of the PCI attempt and the patient was symptom free. Six hours after the interventional procedure, the patient complained of severe chest pain. The ECG demonstrated ST-segment elevation in anterior and lateral leads. Troponin I was mildly elevated but a coronary angiogram did not reveal the impairment of collateral blood flow to the LAD territory. Due to pericarditic chest pain, typical ECG findings and pericardial effusion with elevated C-reactive protein, the diagnosis of acute pericarditis was established, and a course of nonsteroidal anti-inflammatory drugs (NSAIDs) was initiated. Chest pain was relieved and ST-segment elevation almost completely returned to baseline after three days of treatment. The patient was discharged in stable condition without chest pain on the fourth day after symptom onset. Conclusions: Acute pericarditis is a rare complication of PCI. Despite the lack of specific clinical manifestation, post-traumatic pericarditis should be considered in patients with symptoms and signs of pericarditis and a prior history of iatrogenic injury or thoracic trauma.

## 1. Introduction

Acute pericarditis is an inflammatory disease affecting the pericardium with or without the accumulation of excess fluid in the pericardial cavity [1]. Pericarditis causes 0.24% and 0.16% of all cardiovascular admissions in men and in women, respectively [2]. In developed countries, the most frequent origin of pericarditis is viral infection. Rarely, pericarditis can occur after iatrogenic trauma and is known as pericardial injury syndrome [1,3]. Here, we report an atypical case of a patient who developed pericarditis 6 h after an unsuccessful attempt to open a chronic total occlusion (CTO) of the left anterior descending (LAD) artery.

## 2. Case Report

**Figure 1:**
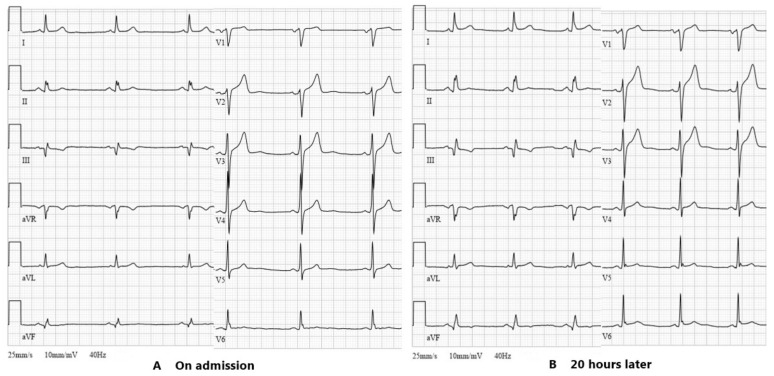
(**A**) Initial ECG of the patient showing sinus bradycardia and Q wave in leads III and aVF. (**B**) ECG after percutaneous coronary intervention with mild ST-segment elevation in anterior (V2–V3) and lateral (I, aVL, V5–V6) leads. ECG, electrocardiogram.

**Figure 2:**
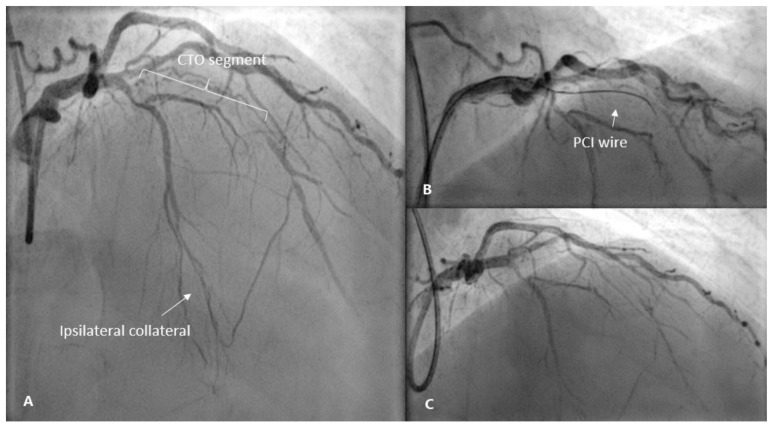
(**A**) Coronary angiogram before PCI showing chronic total occlusion of the LAD and a filling of the mid-distal LAD through ipsilateral collateral. (**B**) Advancement of the wire through the CTO body. (**C**) Post-intervention image with no contrast extravasation. PCI, percutaneous coronary intervention; LAD, left anterior descending; CTO, chronic total occlusion.

A 58-year-old man was admitted to the hospital for an elective percutaneous coronary intervention (PCI) of the chronic total occlusion to the LAD due to exertional angina while on optimal medical therapy. Three months before, due to unstable angina, he underwent coronary angioplasty with the deployment of drug-eluting stents in the right coronary artery. His past medical history included hypertension, dyslipidemia, impaired fasting glycemia and gout. Physical examination as well as the laboratory data involving complete blood count, electrolytes, renal function and hepatic enzymes showed normal findings. An initial electrocardiogram (ECG) revealed sinus bradycardia with a heart rate of 50 beats per minute and pathologic Q-wave in leads III and aVF (Figure 1A). Transthoracic echocardiography demonstrated good systolic function (LV EF 55% without regional wall motion abnormalities) and left atrial enlargement. The attempt to revascularize the coronary artery using the antegrade wire escalation technique (Fielder XTA, Gaia Second and Third wires) was unsuccessful due to the inability to wire the distal true lumen. At the end of the procedure, there was no extravasation of contrast observed (Figure 2C) and the patient was symptom free. A second attempt was planned after one or two months.

**Figure 3:**
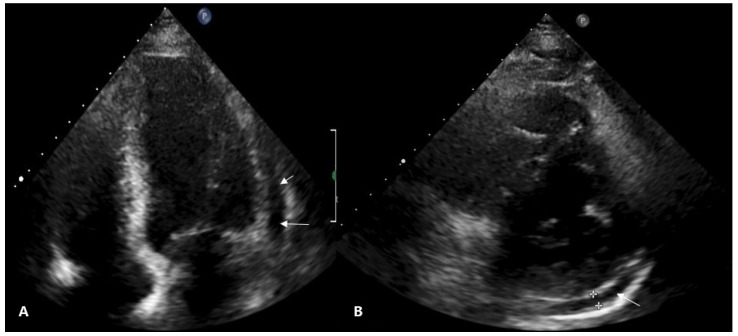
Transthoracic echocardiograms. (**A**) Four chamber view showing small pericardial effusion (5 mm) adjacent to the lateral wall of the left ventricle (arrows). (**B**) 5 mm of pericardial fluid along the inferolateral wall (arrow) in parasternal short axis view.

**Figure 4:**
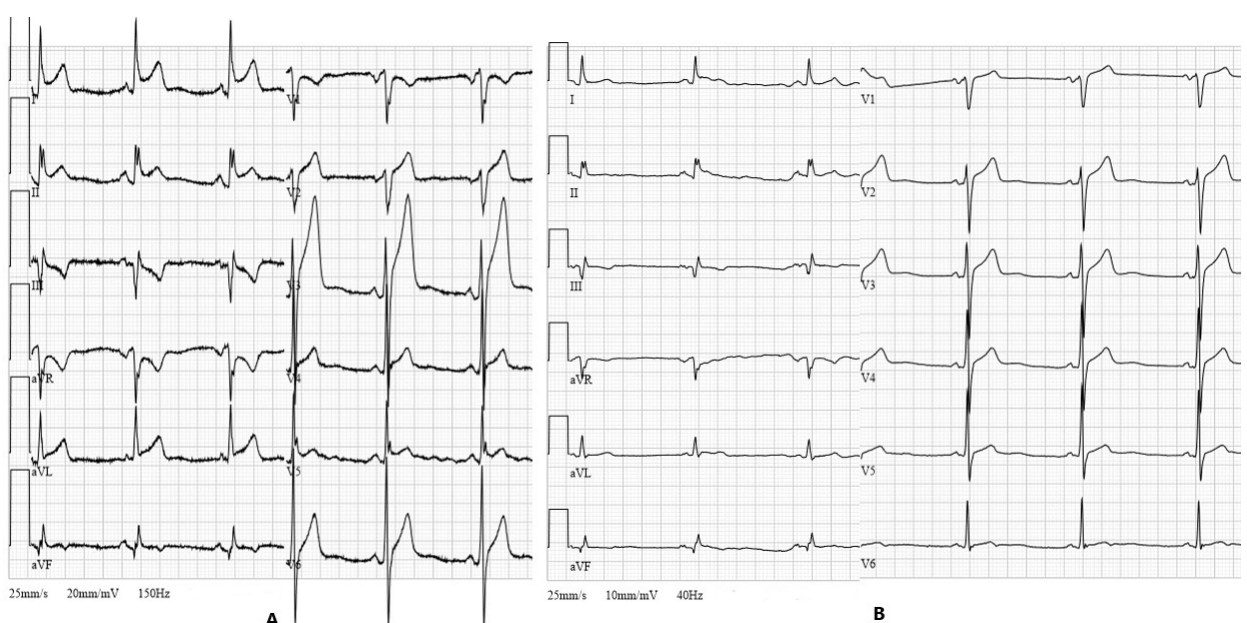
(**A**) ECG demonstrates widespread ST-segment elevation, ST depression in lead aVR and diffuse downsloping depression of PR-segment (36 h after PCI) (20 mm/mV calibration). (**B**) ECG after three days of treatment showing almost complete resolution of the ST-segment elevation (10 mm/mV calibration).

Six hours after the interventional procedure, the patient complained of severe substernal chest pain worsening by bending over and lying on the left side. The ECG demonstrated ST-segment elevation in anterior and lateral leads, suggesting acute anterolateral ST-elevation myocardial infarction (Figure 1B). Troponin I was mildly elevated at 71 ng/L. It was decided to repeat a coronary angiogram in order to evaluate collateral blood flow to LAD as worsening of it could be attributed to the patient’s symptoms and changes on the ECG. Coronary angiogram did not reveal any new findings compared to the previous one, and the collateral flow to the LAD territory was not impaired. An elevated C-reactive protein (CRP) level (98.0 mg/L) with no leukocytosis was observed the next morning. Serum electrolytes and creatinine remained within normal ranges. Transthoracic echocardiogram showed a normal-sized left ventricle with preserved systolic and impaired diastolic functions, left atrial enlargement and hemodynamically non-significant small circumferential pericardial effusion (Figure 3). The diagnosis of acute pericarditis was established and a course of nonsteroidal anti-inflammatory drugs (NSAIDs) was initiated. The patient was given ibuprofen 600 mg tds in addition to aspirin 100 mg od, clopidogrel 75 mg od, a combination of bisoprolol and perindopril 5/10 mg od, amlodipine 5 mg od, moxonidine 0.4 mg od and rosuvastatin 40 mg od. Pantoprazol 20 mg od was also initiated with NSAIDs therapy. The following ECG, which was performed 36 h after PCI, revealed typical ECG findings of pericarditis (Figure 4A).

On the next day, the troponin I level decreased to 37 ng/L and chest pain was significantly relieved. A three-fold reduction in CRP level (from 98.0 mg/L to 30.8 mg/L) was observed after three days of treatment. Repeated ECG showed a return to baseline ST-segment elevation (Figure 4B). In comparison with the previous echocardiogram, no enlargement of pericardial effusion was detected. The patient was discharged in stable condition without chest pain on the fourth day after symptom onset. It was recommended to continue ibuprofen for 12 days three times daily with a reduction in dose after 5 days. Moreover, the patient was given aspirin and clopidogrel, statin and a combination of antihypertensive agents.

**Figure 5:**
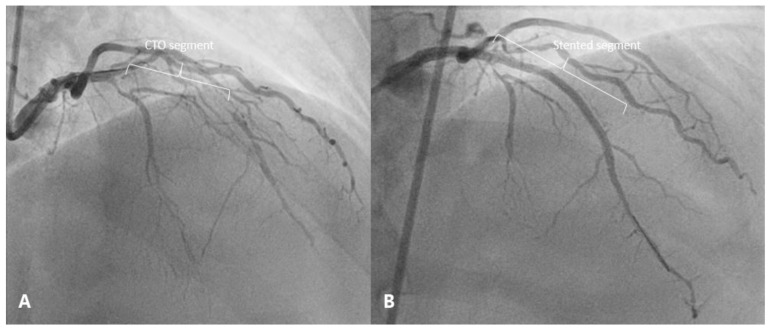
(**A**) Coronary angiogram before PCI showing chronic total occlusion of the LAD. (**B**) Antegrade flow to the distal vascular bed of the LAD after CTO PCI.

The patient had no complaints of pericarditic chest pain after discharge. Two months later, successful PCI to the CTO of LAD was performed using the same antegrade wire escalation technique with an implantation of two drug eluting stents (Figure 5). The patient remained asymptomatic and had no recurrence of pericarditis.

## 3. Discussion

Post-cardiac injury syndrome (PCIS) is a secondary pericarditis resulting from the injury of the pericardium and can be present with or without pericardial effusion. PCIS includes post-myocardial infarction pericarditis, post-pericardiotomy syndrome and post-traumatic pericarditis. The last one can occur after blunt or sharp thoracic trauma and due to an iatrogenic injury [4,5]. Percutaneous coronary intervention, as it was in our case, is known as a possible trigger of pericardial inflammation [6]. Less than 0.2% of PCI procedures are complicated by pericarditis [7].

The pathogenesis of PCIS is not completely understood. It is hypothesized that pericarditis occurs as an immune-inflammatory syndrome due to the injury. Damage to mesothelial cells and blood in the pericardial space initiate an autoimmune response. Released cardiac antigens cause the induction of antibody production. Formed immune complexes deposit in the pericardium and cause inflammation. A latent period lasting weeks to months between the procedure and clinical manifestation is established [4,8]. Taking into account the fact that in our case the first symptoms began 6 h after the procedure, autoimmune pathogenesis due to the second PCI is less possible.

Another explanation of post-traumatic pericarditis development is the extravasation of blood into the pericardial cavity. Blood irritates a visceral layer of the pericardium and causes inflammation [9]. There are some cases describing PCIS due to PCI complicated by coronary perforation or dissection [10,11,12]. The leakage of contrast on X-ray imaging was not observed in our case but pericardial effusion surrounding the heart was present on the echocardiography. We can suppose that the PCI wire could have gone outside the vessel architecture at some point in the CTO-PCI attempt, causing microextravasation not seen during PCI, which could have provoked the synthesis of inflammatory cytokines and fluid accumulation in the pericardial space.

PCIS has no specific diagnostic criteria due to the lack of pathognomonic symptoms and signs. Diagnosis of PCIS should be considered after a cardiac injury when at least two of five criteria are fulfilled: fever without alternative causes, pericarditic or pleuritic chest pain, pericardial or pleural rubs, evidence of pericardial effusion and/or pleural effusion with elevated CRP. ECG can demonstrate diffuse ST-segment elevation with concave morphology and PR depression [1]. The presence of pericardial fluid, its amount and clinical impact on hemodynamics are evaluated by transthoracic echocardiography [5]. The reported patient had typical pericarditic chest pain and met three criteria of PCIS. As ST-segment elevation was present in anterior and lateral walls after unsuccessful PCI and the troponin I level was above the normal reference range, emergent coronary angiogram was performed to rule out myocardial infarction.

The patient received a 15-day course of ibuprofen. According to ESC guidelines, NSAID is an essential treatment of PCIS. For patients with post-myocardial infarction pericarditis and those already taking antiplatelet medications, aspirin is the first choice drug [1]. Ibuprofen can be another option for anti-inflammatory therapy. In a case of the first episode of PCIS, it is recommended to prescribe ibuprofen at 600 mg three times daily over one to two weeks [5]. Similarly to the treatment of acute pericarditis while colchicine is recommended for the prevention of persistent and recurrent pericarditis [13,14], the addition of colchicine to NSAIDs should be considered in cases of PCIS [1]. We have found only one case of post-cardiac injury syndrome after successful PCI of CTO described in the literature. A combination of a high-dose aspirin and colchicine was ineffective. Pleuropericarditis was managed successfully with corticosteroids [8]. 

## 4. Conclusions

Acute pericarditis can occur as a rare complication of percutaneous coronary intervention even during the procedural day. The reported case demonstrated an atypical early onset of PCIS after unsuccessful coronary angioplasty. Post-cardiac injury syndrome should be considered in patients with symptoms and signs of pericarditis and a prior history of percutaneous coronary intervention.

## Data Availability

No new data were created or analyzed in this study. Data sharing is not applicable to this article.
